# Genomic landscape and potential therapeutic targets in alpha-fetoprotein-producing gastric cancer

**DOI:** 10.1007/s10120-025-01594-x

**Published:** 2025-02-10

**Authors:** Likun Zan, Xin Zhang, Lulu Shen, Qi Zhao, Dongfeng Tan, Xiao Peng, Yi Jia, Jiawen Li, Jing Liu, Jiaqi Zhao, Ning Gao, Peng Bu, Yanfeng Xi

**Affiliations:** 1 Department of Pathology, Shanxi Cancer Hospital/Shanxi Hospital Affiliated to Cancer Hospital, Chinese Academy of Medical Sciences/Cancer Hospital Affiliated to Shanxi Medical University, Taiyuan, Shanxi China; 2 Department of Colorectal Surgery, Shanxi Cancer Hospital/Shanxi Hospital Affiliated to Cancer Hospital, Chinese Academy of Medical Sciences/Cancer Hospital Affiliated to Shanxi Medical University, Taiyuan, Shanxi China; 3 Department of Pathology, Heping Hospital Affiliated to Changzhi Medical College, Changzhi, Shanxi China; 4 Departments of Pathology and Medical Oncology, The University of Texas MD Anderson Cancer Center, Houston, TX USA; 5 Department of Pathology, The Third People’s Hospital of Chengdu, The Affiliated Hospital of Southwest Jiaotong University, Sichuan, China; 6 Department of Gastrointestinal Surgery, Shanxi Cancer Hospital/Shanxi Hospital Affiliated to Cancer Hospital, Chinese Academy of Medical Sciences/Cancer Hospital Affiliated to Shanxi Medical University, Taiyuan, Shanxi China; 7 Department of Pathology, Basic Medicine, Shanxi Medical University, Taiyuan, 030001 Shanxi China

**Keywords:** Alpha-fetoprotein-producing gastric cancer, Pathologic features, NGS, Molecular characteristics, Therapeutic targets

## Abstract

Alpha-fetoprotein-producing gastric carcinoma (AFPGC) is a rare and aggressive subtype of gastric cancer (GC). A comprehensive analysis of clinicopathological features, immunophenotypes, molecular characteristics, and survival in AFPGC contributes to identifying potential therapeutic targets and developing new strategies to manage this disease. A retrospective cohort study was conducted at Shanxi Cancer Hospital from January 2018 to December 2020, involving patients diagnosed with GC and elevated AFP serum levels. Among these, 91 patients underwent immunohistochemistry (IHC), fluorescence in situ hybridization (FISH), and next-generation sequencing (NGS) to reveal the immunophenotypic and molecular characteristics of AFPGC. We found that AFPGC is more common in males and primarily occurs in the cardia and antrum of the stomach. A panel of IHC markers including AFP, GPC3, SALL4, CD10, CDX-2, and ATBF1 can be used for the diagnosis and differentiating AFPGC. NGS analysis revealed that *TP53* hypermutation was the most frequent molecular event associated with AFPGC. The altered signaling pathways included disease signal transduction, receptor tyrosine kinase signaling and intracellular second messenger signaling pathways. The cumulative incidence of 21 genes, based on the evidence of clinical actionability in the OncoKB, was found to be 59.34%. Among these genes, *CCNE1*, *ERBB2*, and *EGFR* were the most frequently observed. This underscores the potential benefit of targeted therapy for patients with AFPGC. Furthermore, *LRP1B* and *ARID1A* have been identified as prognostic factors associated with overall survival (OS) and disease-free survival (DFS), respectively. Our results aim to improve AFPGC diagnosis by identifying potential therapeutic targets and prognostic factors, which could help facilitate the development of new treatment strategies.

**Supplementary Information:**

The online version contains supplementary material available at 10.1007/s10120-025-01594-x.

## Introduction

Alpha-fetoprotein (AFP) is a well-established embryonic serum protein, primarily synthesized by embryonic hepatocytes and yolk sac cells. It serves as a significant tumor marker for hepatocellular carcinoma or yolk sac tumors [1]. However, many studies have shown that AFP can also be produced by various other tumors, with gastric cancer (GC) being the most common among them. When GC presents with elevated serum levels of AFP, it is referred to as alpha-fetoprotein-producing gastric carcinoma (AFPGC) [2].

AFPGC is a rare subtype of GC, with an incidence ranging from 1.3% to 15% globally [3]. Compared to other GC types, AFPGC is more aggressive and propensity for liver and lymphatic metastasis, resulting in an extremely poor prognosis. The Cancer Genome Atlas (TCGA) has identified four distinct molecular subtypes of GC: Epstein–Barr virus-infected (EBV), microsatellite instability (MSI), genomically stable (GS), and chromosomal instability (CIN) tumor type [4]. These molecular subtypes, each associated with a specific genomic characterization, provide a foundation for better understanding patient prognosis and tailoring targeted or immunotherapy. However, AFPGC does not clearly fall into any one of these subtypes and lacks specific treatment options.

The pathogenesis of AFPGC remains unclear, and most previous studies were case reports, with few large-scale genomic studies, Genomic characteristics and the potential differences in molecular phenotype among different subtypes have yet to be elucidated [5]. Although molecular targeted therapies and immunotherapy have shown promising efficacy in various cancers, there is no consensus treatment or clearly defined therapeutic targets for patients with AFPGC [6]. Therefore, understanding the molecular characteristics of AFPGC is crucial for developing precise treatment strategies.

In this study, we enrolled 91 patients diagnosed with AFPGC from Shanxi Cancer Hospital. We preformed immunohistochemistry (IHC) and next-generation sequencing (NGS) to analyze the immunophenotypic and molecular biologic characteristics of AFPGC combined with progression-free survival (PFS) and overall survival (OS), aiming to explore potential therapeutic targets and provide referable strategies for its treatment.

## Materials and methods

### Study population

A total of 314 patients diagnosed with GC and elevated serum AFP (> 15 ng/mL) at Shanxi Cancer Hospital from January 2018 to December 2020 were initially included. Of these, 223 patients were excluded due to active hepatitis, liver cirrhosis, hepatocellular carcinoma (HCC), germ cell tumors, or other conditions that could elevate AFP levels. Ultimately, 91 patients who underwent surgical resection with complete clinicopathological data were enrolled. The collected clinicopathological characteristics included the following: gender, age, tumor location, serum AFP value, depth of invasion, tumor size, pTNM stage, lymph node metastasis, nerve invasion, vascular invasion, degree of differentiation, and survival time (calculated from surgery date to the last follow-up or death). The study was approved by the Research Ethics Committee of the Shanxi Cancer Hospital (project number: IIT-2023-020L).

### HE and IHC staining

All specimens were fixed in 3.7% neutral buffered formaldehyde, after dehydration, embedding in paraffin, sectioning, and then stained with hematoxylin and eosin (HE). IHC staining was performed using the EliVision method with the following primary antibodies: rabbit monoclonal antibodies against AFP (Genetech, working solution), GPC3 (Zsbio, working solution), HER-2 (Roche Diagnostics, working solution), CEA (Genetech, working solution), CD10 (Zsbio, dilution 1:200), HNF-1β (Zsbio, working solution), OCT3/4 (Maxim, working solution), p53 (Genetech, dilution 1:300), MLH1 (Zsbio, dilution 1:500), PMS2 (Genetech, dilution 1:70), MSH2 (Zsbio, dilution 1:6000) and MSH6 (Zsbio, dilution 1:100), mouse monoclonal antibody against SALL4 (Genetech, working solution). Rabbit polyclonal antibodies against ATBF1 (Abcam, 1:200) and CLDN6 (Abcam, 1:200) were also used. Sections underwent deparaffinized rehydrated, and antigens were repaired before processing on a Roche automatic immunochemistry instrument. Positive, negative, and blank control slides for comparison. Fluorescence in situ hybridization (FISH, Geneplus) and EBER-ISH were performed according to manufacturer instructions.

AFP and CEA exhibited cytoplasmic positivity, GPC3 and CD10 exhibited cytoplasmic or cell-membrane positivity, HER-2 and CLDN6 exhibited cell-membrane positivity. SALL4, CDX2, ATBF1, HNF-1β, OCT3/4, p53, MLH1, PMS2, MSH2, and MSH6 exhibited nuclear positivity. Five high-power fields (× 400) were randomly selected and evaluated by two experienced pathologists. The IHC score was calculated by multiplying the staining intensity with the positive expression rate, with a score > 3 considered positive. Staining intensity was graded as: 0 for no staining, 1 for light yellow, 2 for brown yellow, and 3 for dark brown. Positive expression rate was categorized as: ≤ 25% (1 point), > 25% to 75% (2 points), and > 75% to 100% (3 points). The interpretation of HER-2 IHC staining results followed the guidelines specified in the GC HER-2 Detection Guidelines (2016 edition). HER-2 positivity was defined as either overexpression (3 +) by IHC or amplification by FISH. Mismatch repair (MMR) deficiency was defined as complete loss of nuclear staining for at least one of the four proteins (MLH1, PMS2, MSH2, or MSH6). P53 overexpression was defined as positive staining in ≥ 70% of tumor cells [7]. Diffuse EBER-ISH labeling indicated EBV-positive GCs.

### Tissue samples sequencing analysis

Genomic DNA was extracted from tumor tissues in resection specimens using the QIAamp DNA FFPE Tissue kit (Qiagen, Valencia, CA), and then the extracted DNA was fragmented and purified. Libraries were prepared using the Oncology Multi-Gene Variant Assay (Geneplus, Beijing, China) and hybridized with custom-designed probes (Roche NimbleGen, Madison, WI, USA) that targeted 1021 cancer-related genes, covering approximately 1.4 Mbp of genomic regions [8–10]. The libraries were quantified using the Qubit dsDNA HS kit (Q32854, Thermo Fisher Scientific). Samples with a DNA library concentration ≥ 20 ng/uL were sequenced on the gene + seq2000 platform (Geneplus, Suzhou, China). The screening of somatic single nucleotide variants (SNV) and small insertions and deletions (InDels) was executed using the MuTect (v1.1.4) and GATK (v3.4–46-gbc02625) software (both Broad Institute). Copy number variations (CNV) and structural variations (SV) were identified using Contra (v2.0.8) and NoahCare structural variations detection (in-house), respectively [11–13]. Tumor mutational burden (TMB) was calculated as the total number of mutations in coding regions (including synonymous and non-synonymous point mutations, substitutions, insertions, and deletions) [14], and dichotomized by the median value [15]. Molecular typing was performed according to the TCGA classifications for gastric carcinoma, as well as MMR status, EBV presence, and all gene mutation information [4].

### Prognosis and follow-up

Patients who were enrolled in this study were followed up through outpatient examinations or telephone consultations to assess health status, tumor recurrence, and survival until December 31, 2022. OS and DFS were calculated from surgery completion to death/last follow-up and disease recurrence/ death, respectively.

### Statistical analysis

Data were analyzed using SPSS software (version 27.0; SPSS, Inc.). Categorical variables were compared using the Chi-square test and/or Fisher exact test. The optimal serum AFP cut-off for predicting prognosis was determined using receiver operator characteristic curve (ROC) analysis. Statistical analysis of gene mutation and survival was performed using R language software (version 4.3.1). Survival analysis was performed using the Kaplan–Meier (KM) method with a 95% Confidence Interval (CI). Cox multivariate regression analysis was employed to explore prognostic factors among patients, considering a significance level of *P* < 0.05 from the KM analysis. Mutations among different histologic subtypes were identified, and Kyoto Encyclopedia of Genes and Genomes (KEGG) pathway enrichment analysis was conducted. All tests were two-sided with significance set at P < 0.05.

## Results

### Clinicopathological characteristics

**Fig. 1:**
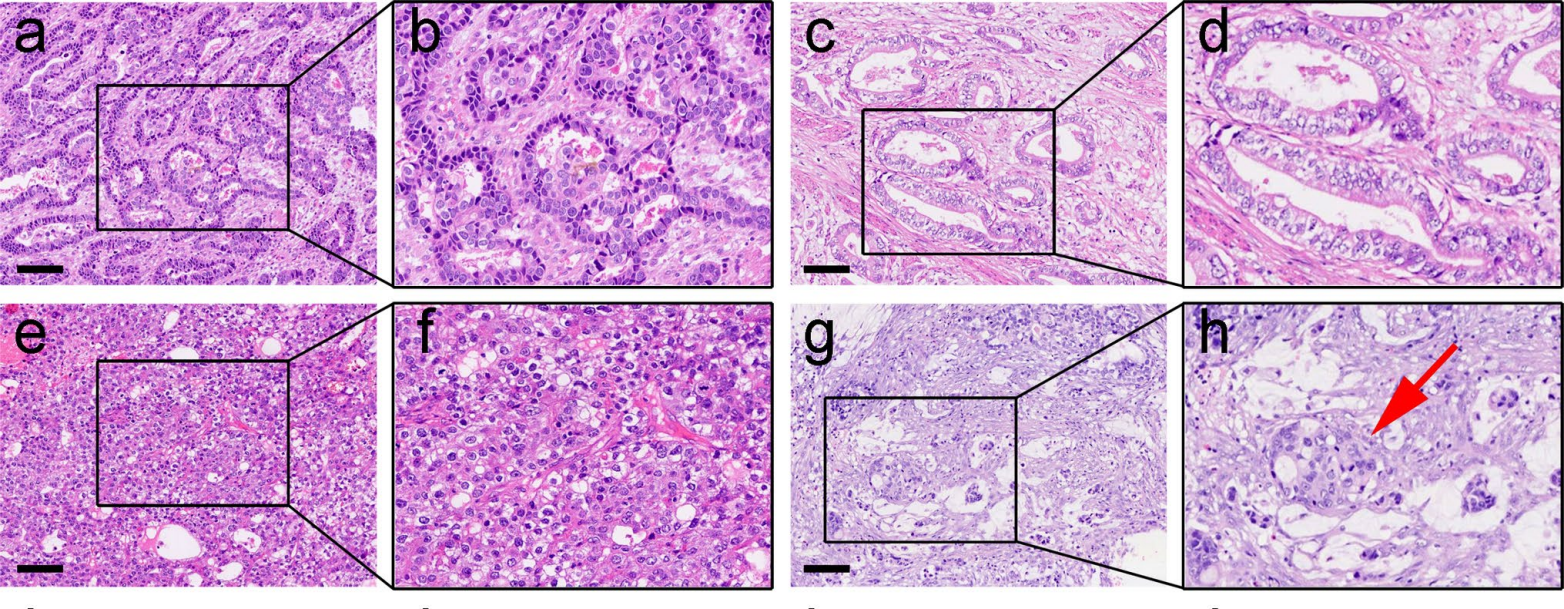
HE staining of AFPGC four histologic subtypes. **a**, **b** Common intestinal adenocarcinoma type (COM). **c**, **d** Enteroblastic type (ENT). **e–f**: Hepatoid type (HPT). **g**, **h** Yolk sac tumor type (YST). Red arrow: Glom-like bodies (Shiller-Duval bodies). Box: to be enlarged. Bar = 100 μm

Clinical characteristics of 91 AFP-producing GC patients enrolled in this study are summarized in Table 1 (Supplementary). AFPGC is more common in the male population, with 73 males and 18 females, for a male-to-female ratio of 4.05:1. The age of this cohort ranged from 27 to 82 years. All 91 patients had elevated serum AFP levels preoperation, ranging from 16.17 to 60,500.00 ng/ml. At the tumor site, the AFPGC were mainly located in the cardia (41/91) and the gastric antrum (31/91), followed by the gastric body (14/91) and the esophagogastric junction (5/91). According to the depth of invasion, five cases invaded the mucosa and submucosa (T1), ten invaded the muscularis propria (T2), 70 invaded the subserosa (T3), four invaded the serosal layer but did not invade adjacent organs (T4a), and two invaded the serosal layer and invaded adjacent organs (T4b). The maximum diameter of the tumors ranged from 2.00 cm to 19.00 cm. Our cohort was classified into four histologic subtypes: (1) Common intestinal adenocarcinoma type (COM): This subtype accounted for 35 cases (38.46%) and including poorly, moderately, and well-differentiated types, exhibiting tubular and papillary structures (Fig. 1a, b). (2) Enteroblastic type (ENT): Consisting of 12 cases (13.19%) with clear cytoplasm composed of cuboidal or columnar cells resembling primitive intestinal cells, and forming tubular, papillary, or adenoid structures. (Fig. 1c, d). (3) Hepatoid type (HPT): Observed in 40 cases (43.95%), characterized by cells with clear cytoplasm or eosinophilic granules arranged in medullary or cord-like structures with vesicular nuclei, prominent nucleoli, reminiscent of liver tissue organization (Fig. 1e, f). (4) Yolk sac tumor type (YST): Identified in 4 cases (4.40%), exhibiting a network-like arrangement of interconnected cytoplasm with communicating cavities and cysts resembling a labyrinth structure. (Fig. 1g, h). In addition, we also found a small proportion of other types of adenocarcinoma components in 91 cases of AFPGC, with two cases exhibited mixed ENT and HPT components, three displayed mucinous adenocarcinoma components, and three combined with neuroendocrine carcinoma. All mixed components accounted for less than 10%. No statistical differences were observed in gender, age, serum AFP level, tumor location, depth of invasion, pTNM stage, tumor size, lymph node metastasis, nerve invasion, vascular invasion, and differentiation degree among the histologic subtypes (Table 1).

### Expression of AFPGC immunohistochemical markers

**Fig. 2:**
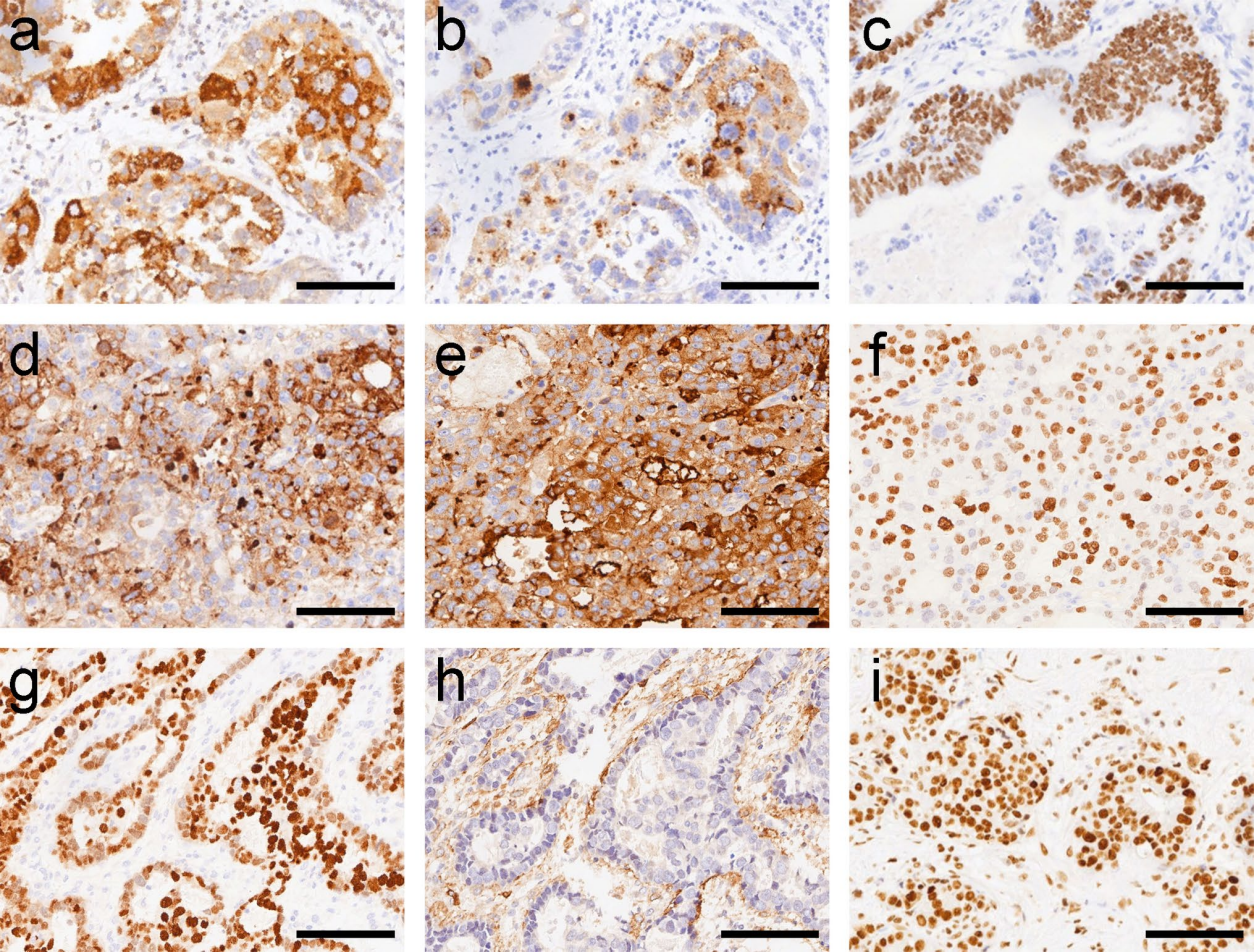
Immunohistochemical phenotyping of AFPGC. **a**, **b**, **c** AFP, GPC3, and SALL4 positive expressed in ENT. **d**, **e**, **f **AFP, GPC3, and SALL4 positive expressed in HPT. **g**, **h** CDX2, CD10 positive expressed in COM. **i** HNF-1β showed nuclear positive in YST. Bar = 100 μm

We conducted IHC in this cohort to examine the expression of markers associated with the four subtypes. The IHC results revealed that the embryonic proteins of AFP (Fig. 2a, d), GPC3 (Fig. 2b, e), and SALL4 (Fig. 2c, f) expressed in AFPGC were 51.65%, 64.84%, and 58.24%, respectively. We found that the expression of the above three embryonic proteins in HPT was significantly higher than those in the other three subtypes (*P*<0.05). Moreover, there was a significant correlation between their expression and patients' serum AFP level (*P*<0.05). The expression of AFP was significantly positively associated with vascular invasion (*P*<0.05). The intestinal epithelial differentiation markers CDX-2 (Fig. 2g) and CD10 (Fig. 2h) were expressed in 85 (93.41%) and 81 (89.01%) cases of AFPGC patients, respectively. Meantime, we found CEA was expressed in 84 (92.31%) cases which were significantly correlated with lymph node metastasis (*P*<0.05).

In addition, CLDN6 was expressed in 88 (96.70%) cases, while OCT3/4 was only expressed in 3 (3%) cases. Notably, OCT3/4 showed high expression specifically in ENT. We also found ATBF1 and HNF-1β (Figure 2i) expressed in 22 (24.18%) and 78 (85.71%) patients of AFPGC, respectively. Notably, the negative rate of ATBF1 was higher in the HPT subtype compared to other subtypes (*P* <0.05). The prevalence of mutant p53 was observed in 75 cases (82.42%), while p53 wild-type was in 16 cases (17.58%). Patients aged ≥60 years with mutant p53 exhibited significantly higher compared to those with p53 wild-type. Furthermore, 78 cases (85.71%) displayed proficient MMR (pMMR) and 13 (14.29%) as deficient MMR (dMMR, Table 1**,** Supplementary). Two cases exhibiting HER-2 (3+), and eight with HER-2 (2+) expression, which were further examined by FISH. The results indicated that five out of eight HER-2 (2+) cases with HER-2 amplification (Supplementary Figure 1). Only one AFPGC with diffuse EBER-ISH labeling was considered EBV-positive GC.

### Mutations and signatures of AFPGC

**Fig. 3:**
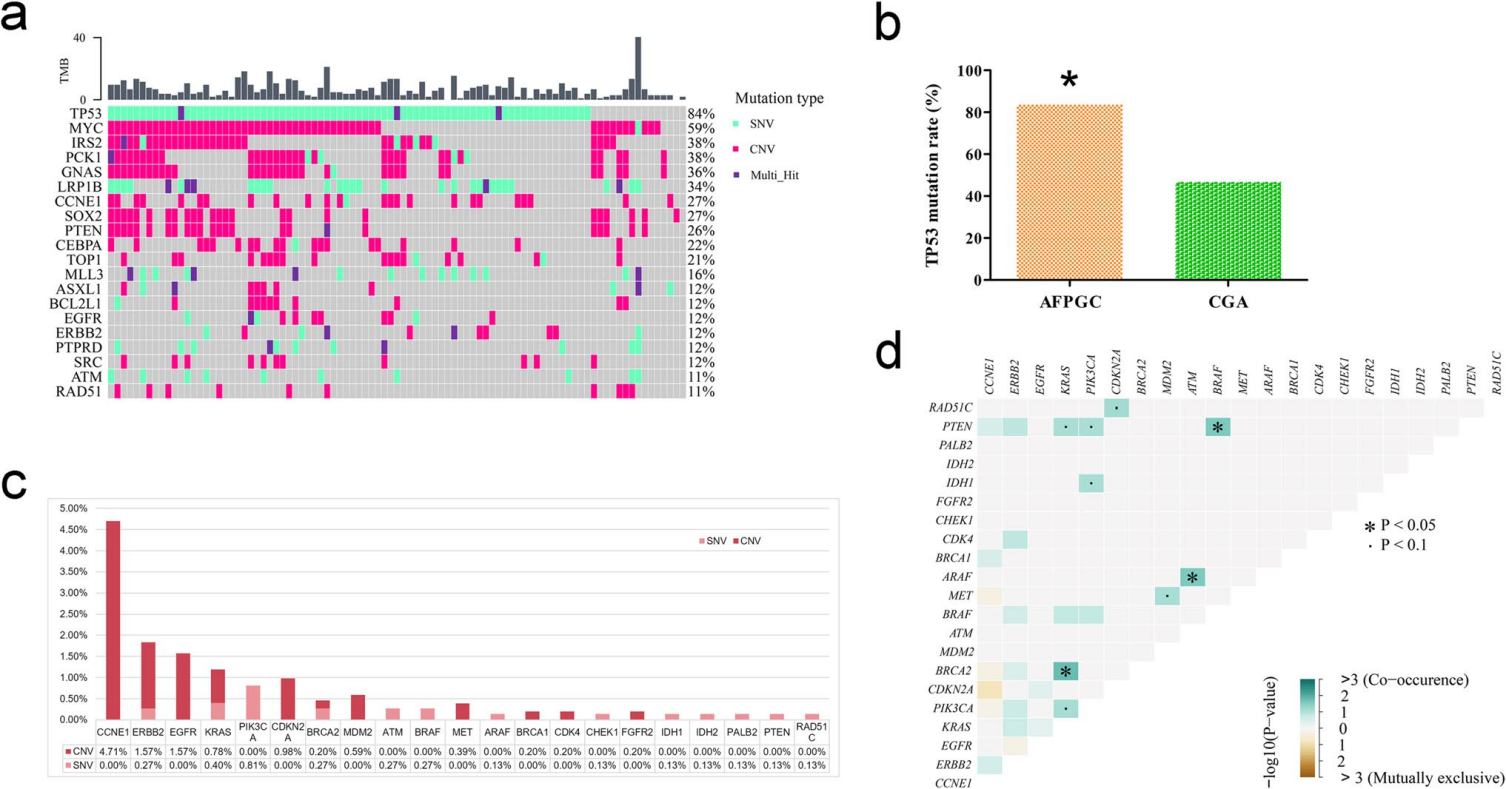
Mutations signatures and potential therapeutic targets of AFPGC. a: TMB and Top 20 somatic mutations of 91 AFPGC tissues. **b** The *TP53* mutation rate in AFPGC and CGA. *P < 0.05. **c** Comparative analysis of the approved target genes of drugs in the OncoKB website. d: Mutually exclusive co-mutations in 21 target genes. *P < 0.05; ▪*P* < 0.1

Next, we performed NGS methods to investigate the mutational characteristics of 91 AFPGC patients in this study, with a total of 1271 variants successfully detected, including 744 (58.54%) SNVs, 510 (40.13%) CNVs, and 17 (1.34%) SVs, with a mean number of variants of 14. The vast majority of harbored at least one mutation (89/91, 97.8%), and only two cases tested negative. The most frequently altered genes in AFPGC were *TP53* (84%), *MYC* (59%), *IRS2* (38%), *PCK1* (38%), and *GNAS* (38%). Other mutation frequencies in more than 10% were *LRP1B* (34%), *CCNE1* (27%), *CEBPA* (22%), *MLL3* (16%), *EGFR* (12%) and *ERBB2* (HER-2, 12%). In addition, TMB values were also analyzed, with a median of 5.76 muts/Mb (Fig. 3a).

Then, we analyzed different mut-types, starting with the SNVs analysis showed *TP53* mutation was the most frequent (76/91, 83.52%), followed by *LRP1B* (31/91, 34.07%), *MLL3* (15/91, 16.48%), *PTPRD* (11/91, 12.09%), *EPHA3* (9/91, 9.89%), *ATM* (9/91, 9.89%), and *APC* (7/91, 7.69%) (Fig. 3a). The frequencies of *TP53* mutation in AFPGC were significantly higher than that in common gastric adenocarcinoma (CGA) from the Cancer Genome Atlas database [4] with rates of 83.52% (76/91) versus 46.77% (138/295, *P* < 0.05, Fig. 3b). Second, we found that CNVs were observed in 80 patients. However, most CNVs involve more than two genes occurring simultaneously (68/80, 85%). The most frequently gene amplification included *GNAS* (32/91, 35.16%), *PCK1* (31/91, 34.07%), *IRS2* (31/91, 34.07%), *CCNE1* (24/91, 26.37%), *PTEN* (24/91, 26.37%), *CEBPA* (19/91, 20.88%), *TOP1* (17/91, 18.68%), *SRC* (11/91, 12.09%), *BCL2L1* (10/91, 10.99%), *VEGFA* (10/91, 10.99%), *EGFR* (8/91, 8.79%) and *ERBB2* (7/91, 7.69%). Corresponding to amplification, the most common gene deletions were *MYC* (53/91, 58.24%) and *SOX2* (25/91, 27.47%) (Fig. 3a). Third, SV mutation was detected in 16 patients (≥ 1 SVs), with one patient having SVs in both the *BRCA1-LOC100507425* and *MET* genes. The rest have one gene SV involved the following genes: *AKAP9-BRAF*, *IRF2BP1-MYC*, *C14orf177-GNAS*, *PDGFRA* rearrangement, *MAGT1-ATRX*, *EPHA6-ROS1*, *FANCM-GRIA4*, *PREX2-ROS1*, *FGFR3-TACC3*, *EPHA6-ROS1*, *EGFR* rearrangement, *FGFR2-UBE2D1*, *ERC1-BRAF*, *RPS6KA4-BRCA1*, *AIM1-FGFR3*.

### Potential therapeutic targets

To further explore the potential therapeutic targets of AFPGC, we conducted a comparative analysis of the approved target genes of drugs in the OncoKB (OncoKB™—MSK's Precision Oncology Knowledge Base) website. We found the cumulative incidence of 21 genes was 59.34%, of which CCNE1, ERBB2, and EGFR were the highest (Fig. 3c). The genes associated with targeted drugs were often co-occurrence (Fig. 3d).

Therewith, we compared the differences in molecular characteristics between different histologic subtypes and between subgroups with or without metastasis. The results showed that the frequencies of *CREBBP* and *FGF14* in the yolk sac type were significantly higher than those in other subtypes. We also found that the incidence of *CEBPA* mutation was more likely to occur in patients with metastasis, whereas *MLL3* mutation was more likely to occur in patients without metastasis.

### Pathway enrichment analysis

**Fig. 4:**
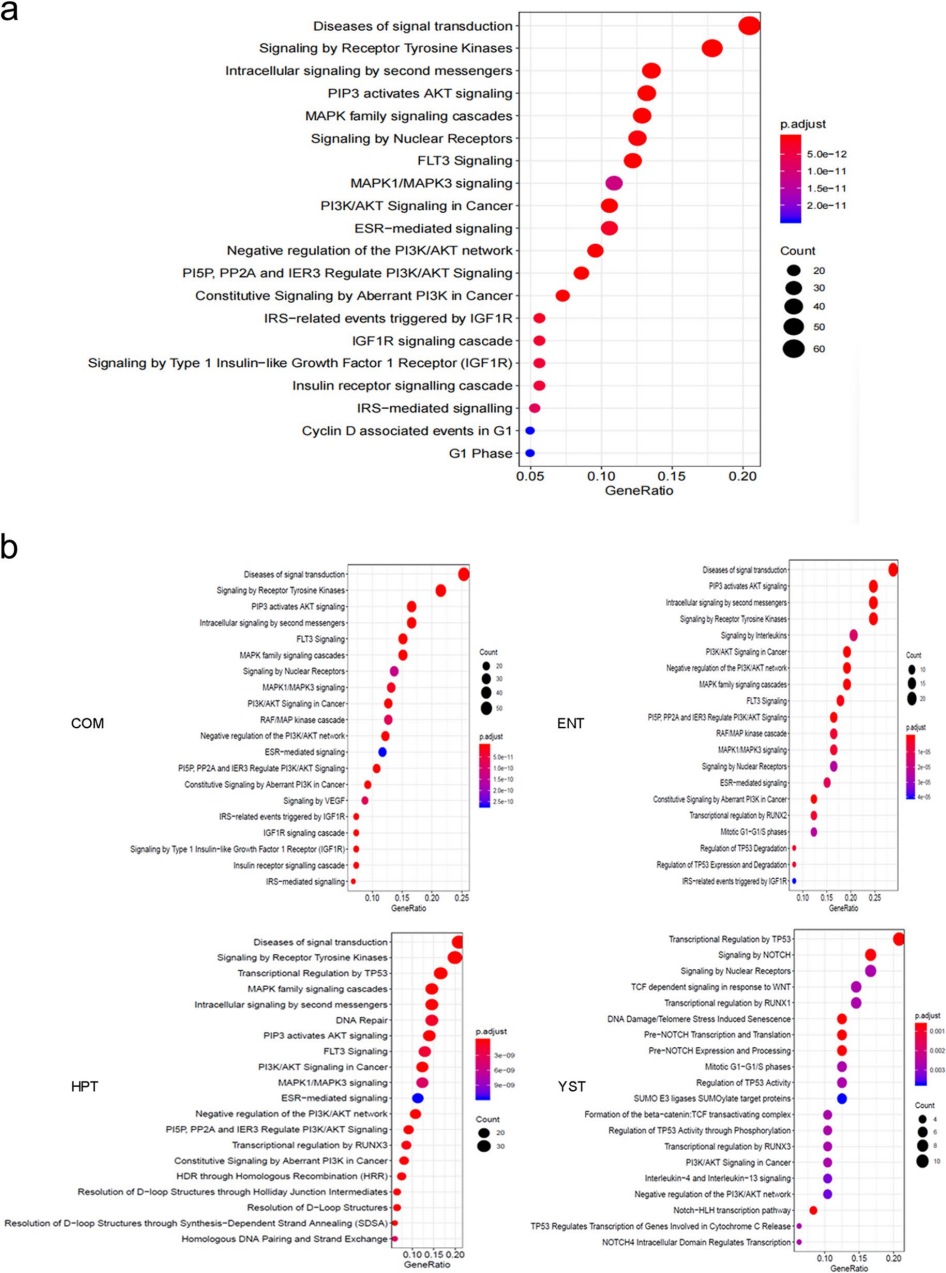
KEGG pathway enrichment analysis of AFPGC. **a** AFPGC patients. **b** Patients of COM, ENT, HPT and YST subtypes

To further clarify whether these genomic alterations lead to differences in cancer-related signaling pathways, we performed pathway enrichment analysis using the KEGG database (Fig. 4). The results revealed several signaling pathways were enriched, including disease signal transduction, receptor tyrosine kinase signaling, intracellular second messenger signaling, PIP3-Akt signaling pathway, MAPK family signaling cascade, nuclear receptor signaling, FLT3 signaling, MAPK1/MAPK3 signaling, PI3KI-AKT Signaling as well as ESR-mediated signaling. We found that these signaling pathways were mainly enriched in pathways related to cell proliferation, invasion, and metastasis. However, no significant differences between histologic subtypes were observed **(**Fig. 4b**)**.

### Survival analyses of AFPGC

**Fig. 5:**
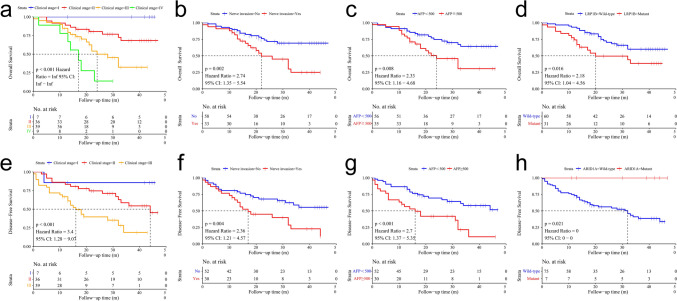
Kaplan–Meier curves for overall survival (OS) and Disease-Free Survival (DFS). a-g: Patients with high clinical stage, nerve invasion, serum AFP ≥ 500 ng/ml, and *LRP1B* mutation had poor OS. **e**–**h**. Patients with high clinical stage, nerve invasion, serum AFP ≥ 500 ng/ml, and *ARID1A* mutation had poor DFS

Our next step aims to conduct a ROC curve to identify the optimal cut-off points of serum AFP values for survival analysis. As shown, 510 ng/ml was the optimal cut-off, the sensitivity was 52.8%, the specificity was 72.7%, and the area under the curve was 0.628 (*P* = 0.039) (Supplementary Fig. 2). Therefore, we chose “cut-off = 500 ng/ml” to perform the follow-up analysis. The results indicate that patients with serum AFP ≥ e500 ng/ml had poor prognosis. The analysis of OS included 91 cases, a univariate Cox analysis based on OS as clinical outcomes revealed that clinical stage, venous invasion, nerve invasion, distant metastasis, serum AFP, and *LRP1B* mutation were prognostic factors for AFPGC (*P* < 0.05), Furthermore, a multivariate Cox proportional hazards regression model revealed that clinical stage and nerve invasion were still independent prognostic factors (*P* < 0.05, Fig. 5, Supplementary Fig. 3, Table 2). The DFS analysis included 82 patients who underwent a radical operation. A univariate Cox analysis based on PFS as clinical outcomes revealed that tumor size, lymph node-positive, clinical stage, venous invasion, nerve invasion, serum AFP, *ARID1A* mutation were prognostic factors for AFPGC (*P* < 0.05). In the multivariate Cox proportional hazards regression model, clinical stage, serum AFP and nerve invasion were still independent prognostic factors (*P* < 0.05, Fig. 5, Supplementary Fig. 3, Table 3).

To further explore the influence of molecular characteristics on prognostic, we analyzed the relationship between different gene mutations and survival. We found that the *LRP1B* was a risk factor for OS, while *ARID1A* acted as a protective factor for DFS. Subsequently, we analyzed the effects of mutations on OS and DFS with mutation frequencies greater than 8%. The results indicated that patients with the *LRP1B* mutation experienced significantly decreased OS (*P* = 0.016, HR = 2.18, 95% CI: 1.04–4.56), while patients carried *ARID1A* mutation with significantly increased DFS (*P* = 0.021).

### Molecular typing analysis in AFPGC

**Fig. 6:**
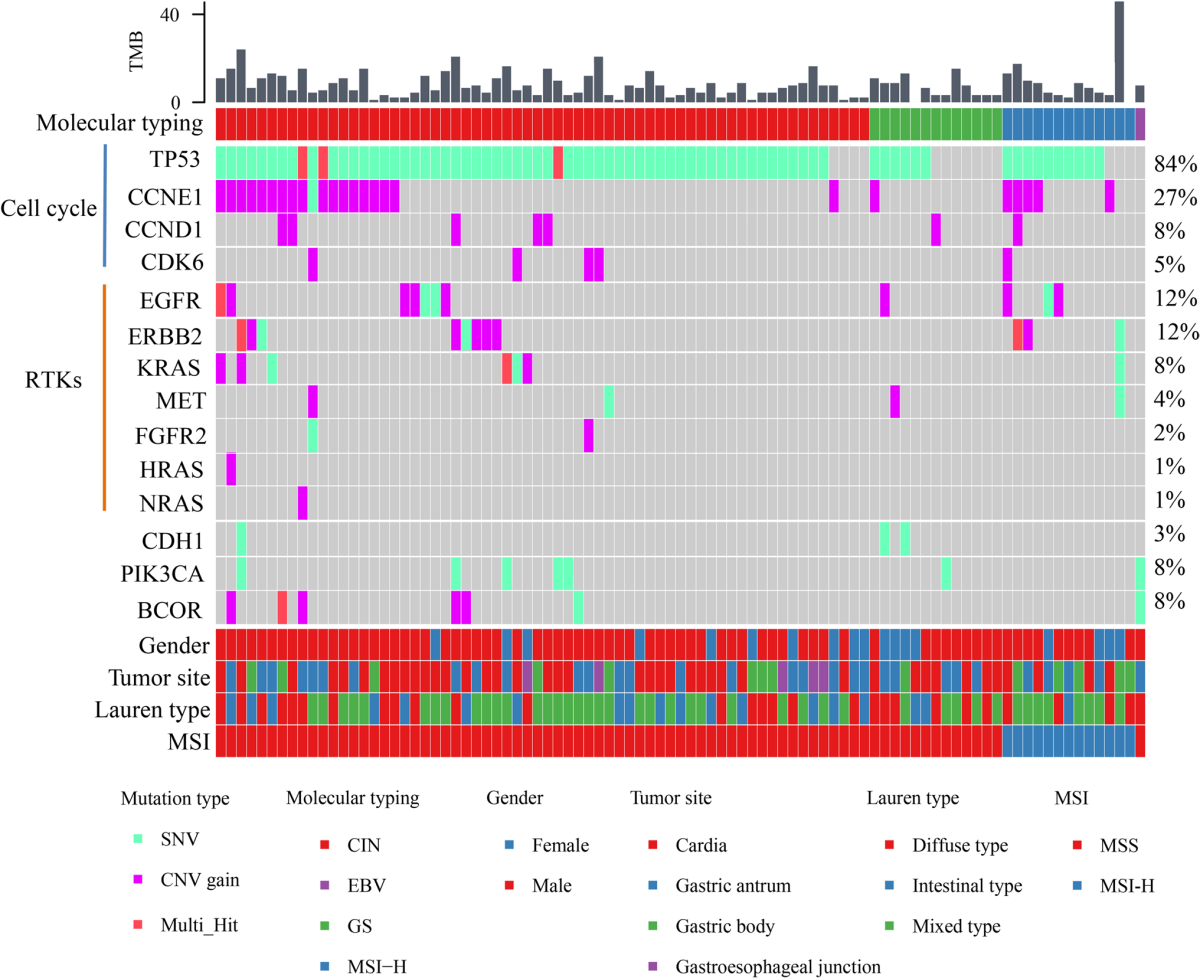
The mutations landscape of AFPGC

Based on the molecular characteristics of TCGA typing of GC [4], we typed the patients in our cohort (Fig. 6). A total of 64 patients (70.33%) were classified as CIN type, primarily occurring in cardia and antrum, and most of them were diffuse type in Lauren’s classification. The mutation frequency of TP53 in CIN type was as high as 93.75%, and chromosome amplification was found in cell cycle regulatory genes (*CCNE1*, *CCND1*, *CDK6*) and some RTKs genes (*EGFR*, *ERBB2*, *FGFR2*, *MET*, *RAS*). 13 patients (14.29%) were classified as MSI type, the predilection location of which were gastric antrum and body, and most of them belong to mixed type in Lauren’s classification. The typical molecular events were *PIK3CA*, *ERBB2*, and *EGFR* mutation, along with some gene chromosome amplification. Only one patient (1.1%) was EBV-positive GC, which also occurred in the gastric antrum and belonged to the diffuse type in Lauren’s classification. Notably, there were no *CDKN2A* mutation, compared to the TCGA cohort. 13 patients (14.29%) were classified as GS type, the frequency of *CDH1* (15.38%) mutation was high in this type, and *TP53* mutation frequency was lower than CIN type. Finally, we performed a survival analysis for different molecular subtypes, and the results showed that the prognosis of GS was the worst (Fig. 6).

## Discussion

AFPGC, first described by Bourreille in 1970, is a rare and aggressive GC characterized by elevated serum AFP or positive AFP IHC staining in tissues [16]. AFPGC is associated with a high incidence of liver metastasis and poor prognosis [17]. Despite its clinical importance, comprehensive research on the clinicopathological and molecular characteristics of AFPGC is lacking, and there is no consensus on diagnostic criteria or standard treatment guidelines. In this study, we defined AFPGC as preoperative serum AFP exceeding 15 ng/mL and found that patients with serum AFP levels above 500 ng/mL had a poorer prognosis. AFPGC most commonly occurs in males aged 27 to 82 years, primarily in the cardia (41/91) and antrum (31/91) of the stomach, with clinical manifestations similar to CGA. AFPGC is classified into four distinct histologic subtypes, namely COM, ENT, HPT, and YST [17, 18]. The COM subtype is the most likely to be misdiagnosed because of no specific morphologic characteristics, suggesting that routine detection of serum AFP in patients with GC is necessary.

We confirmed the utility of the panel of IHC markers-AFP, GPC3, and SALL4-in distinguishing AFPGC from CGA [19]. Specifically, the AFP, GPC3, and SALL4 were positively expressed in 51.65%, 64.84%, and 58.24% of AFPGC cases, respectively. Atotal of 71(78.02%) patients expressed at least one of those markers, indicating that their combined use could improve diagnosis accuracy in this study. We also found that the HPT subtype showed particularly high expression of these embryonic proteins, potentially explaining the elevated serum AFP levels often observed in this subtype. Furthermore, SALL4-positive patients exhibit an enhanced propensity for metastasis [20], and CEA-positive correlated with a higher lymph node metastasis rate [21]. In addition, the high expression of CDX-2 and CD10 in the tubular and papillary areas of AFPGC suggests intestinal epithelial differentiation in the ENT subtype [22, 23]. As an inhibitory transcription factor of AFP, ATBF1 can interact with liver activation protein (LAP) and enhancer binding protein (C/EBP-β) to inhibit the production and secretion of AFP [24]. Our study also indicated a negative correlation between ATBF1 expression and AFP levels. The roles of ATBF1 in AFPGC require further research.

Recently, the TCGA divided GC into four subtypes: EBV-positive, MSI, GS and CIN [4]. Meanwhile, the Asian Cancer Research Group (ACRG) classified GC into four types: mesenchymal, MSI, tumor protein p53 (TP53) active, and TP53 inactive [25]. These classifications provide valuable information for individualized treatment and stratification of patients. However, AFPGC does not fit neatly into any of these subtypes, indicating that AFPGC may possess unique molecular biologic characteristics at the genomic level. Our NGS results revealed that the Top ten mutations are *TP53*, *MYC*, *IRS2*, *PCK1*, *GNAS*, *LRP1B*, *CCNE1*, *SOX2*, *PTE* and *CEBPA* in AFPGC. The frequency of *TP53* was 83.52% in AFPGC, which was significantly higher than that in CGA (46.77%). Meantime, *GNAS* and *PCK1* were identified as the most common amplification, while *MYC* and *SOX2* were the most frequent deletions. *GNAS* mutations occur in a variety of cancers [26], when *GNAS* is activated, the cAMP pathway becomes linked to the RAS-RAF-MEK-ERK signaling pathway, cell proliferation and angiogenesis in appendiceal cancers [27]. *MYC* is a crucial protein in tumor progression, regulating proliferation and apoptosis [28]. The phosphorylation of *MYC* is partly controlled by the major AFPGC pathway-receptor tyrosine kinase /phosphatidylinositol 3-kinase /Akt pathway /mTOR signaling [29]. This suggests that *MYC* mutation may lead to abnormal cell proliferation and apoptosis, thereby accelerating the progress of AFPGC.

In this study, we conducted the KEGG enrichment analyses, which revealed that disease signal transduction and receptor tyrosine kinase signaling pathways are significantly enriched. This finding suggests their involvement in AFPGC development and potential therapeutic targets. Meantime, recent research have indicated that disease signal transduction pathways may be involved in chemotherapy resistance in AFPGC [30, 31]. In recent years, molecular targets such as EGFR, C-MET, and FGFR2 of the receptor tyrosine kinase family have garnered attention in GC, due to their ability to activate cascades of signal transduction pathways involved in cell differentiation [32, 33]. As one of the most enriched signaling pathways, receptor tyrosine kinase signaling plays a significant role in the occurrence and development of AFPGC. This result implies that patients with AFPGC may benefit from targeted therapy of genes associated with receptor tyrosine kinase signaling.

To further explore potential therapeutic targets for AFPGC, we compared our findings with variants corresponding to drugs approved by OncoKB. In our cohort, a total of 21 gene mutations associated with targeted therapies were detected in 59.34% of patients, with *CCNE1*, *ERBB2*, and *EGFR* mutations being the most frequently observed. This highlights the potential benefit of targeted therapy for AFPGC patients and emphasizes the importance of genetic testing. In addition, we found that *CEBPA* mutation was associated with metastasis, whereas *MLL3* mutation was more common in non-metastatic cases. This finding suggests potential roles for these genes in AFPGC metastasis and warrants further validation.

AFPGC always presents lymphatic vessel invasion, vascular invasion, lymph node and liver metastasis, and high tumor budding [34]. In this study, the incidence of vascular invasion, nerve invasion, and lymph node metastasis of AFPGC was 59.34% (54/91), 36.26% (33/91), and 71.43% (65/91), respectively. Furthermore, the incidence rate of liver metastasis was 19.78% (18/91), significantly higher than 6% observed in CGA. It has been demonstrated that liver metastasis serves as the primary cause of mortality among AFPGC patients [35]. Consistent with previous reports [36], serum AFP level is a prognostic factor for OS, we found that AFPGC patients with serum AFP ≥ 500 ng/mL had a poor prognosis. Our multivariate analysis identified serum AFP as an independent prognostic factor for both OS and DFS, indicating that serum AFP is an important predictor of prognosis for AFPGC. Therefore, routine serum AFP detection is essential in GC.

Finally, we identified *LRP1B* mutation as a risk factor for OS and *ARID1A* mutation as a protective factor for DFS in AFPGC. These findings indicated a potential prognostic value for these mutations. *LRP1B* can activate multiple carcinogenic pathways and enhance cellular activity, potentially contributing to the poor prognosis observed in hepatocellular carcinoma, non-small cell lung cancer, ovarian clear cell carcinoma, and other tumors [37, 38]. However, the molecular mechanisms of *LRP1B* in patients with AFPGC need further investigation. The *ARID1A* protein complex is a highly conserved, ATP-dependent chromatin remodeling complex. The loss of *ARID1A* results in the destruction of the SWI/SNF complex, leading to an imbalance of genes expression in cell stemness, and cell differentiation, and promotes the growth of cancer [39]. *ARID1A* mutations are frequent in both EBV and MSI GC subtypes [40, 41]. Some studies showed that the loss of *ARID1A* expression in GC patients has been significantly associated with poor survival [42]. However, given the rarity of AFPGC and the limited case studies available, these findings suggest that further validation with larger cohort research is required. In addition, some studies have also demonstrated that loss expression of *ARID1A* is significantly correlated with PD-L1 positive expression, which indicates that patients may respond more effectively to immune checkpoint inhibitors (ICIs). Therefore, *ARID1A* may become a biomarker for GC immunotherapy in future.

In conclusion, our study enrolled 91 patients with AFPGC and conducted a comprehensive analysis of its clinicopathological, immunophenotypic, and molecular characteristics. We uncovered that a combination of 21 genes could improve the diagnosis of this aggressive GC subtype. In addition, we systematically reported the landscape of genetic alterations associated with AFPGC for the first time, to the best of our knowledge. Our findings revealed an enrichment of receptor tyrosine kinase signaling, which is a critical pathway related to targeted therapy, along with two significant prognostic targets, *LRP1B* and *ARID1A*. The above results provide a comprehensive overview of AFPGC and support further development of potential therapeutic targets.

## Supplementary Information

Below is the link to the electronic supplementary material.

Supplementary file1: FISH of AFPGC showed Her-2 amplification. Bar = 20 μm (TIF 634 KB)

Supplementary file2: ROC Curve of serum AFP values (TIF 498 KB)

Supplementary file3: Kaplan–Meier curves for overall survival (OS) and Disease-Free Survival (DFS). a, b: Patients with venous invasion, and metastasis had poor OS. c–e: Patients with venous invasion, lymph node metastasis, and tumor size ≥ 5 cm had poor DFS (TIF 13908 KB)

Supplementary file 4 (DOCX 28 KB)

Supplementary file 5 (DOCX 21 KB)

Supplementary file 6 (DOCX 21 KB)

## Data Availability

The datasets used and/or analyzed during the current study are available from the corresponding author on reasonable request.
