# Supplementary figures and images for: Genomic landscape and potential therapeutic targets in alpha-fetoprotein-producing gastric cancer
Source: Gastric Cancer. 2025 Feb 10;28(3):372–83. doi: 10.1007/s10120-025-01594-x (PMC11993487; doi:10.1007/s10120-025-01594-x)

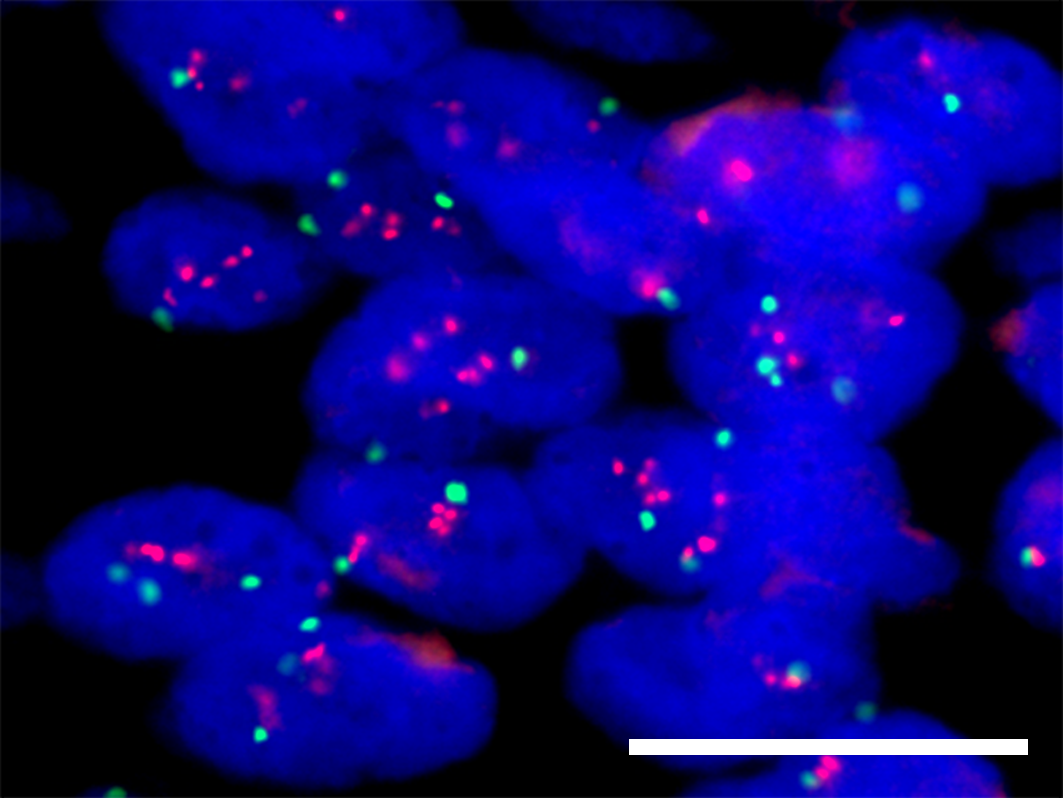

Supplement: Supplementary file 1 — Supplementary file1: FISH of AFPGC showed Her-2 amplification. Bar = 20 μm (TIF 634 KB) [file 10120_2025_1594_MOESM1_ESM.tif]

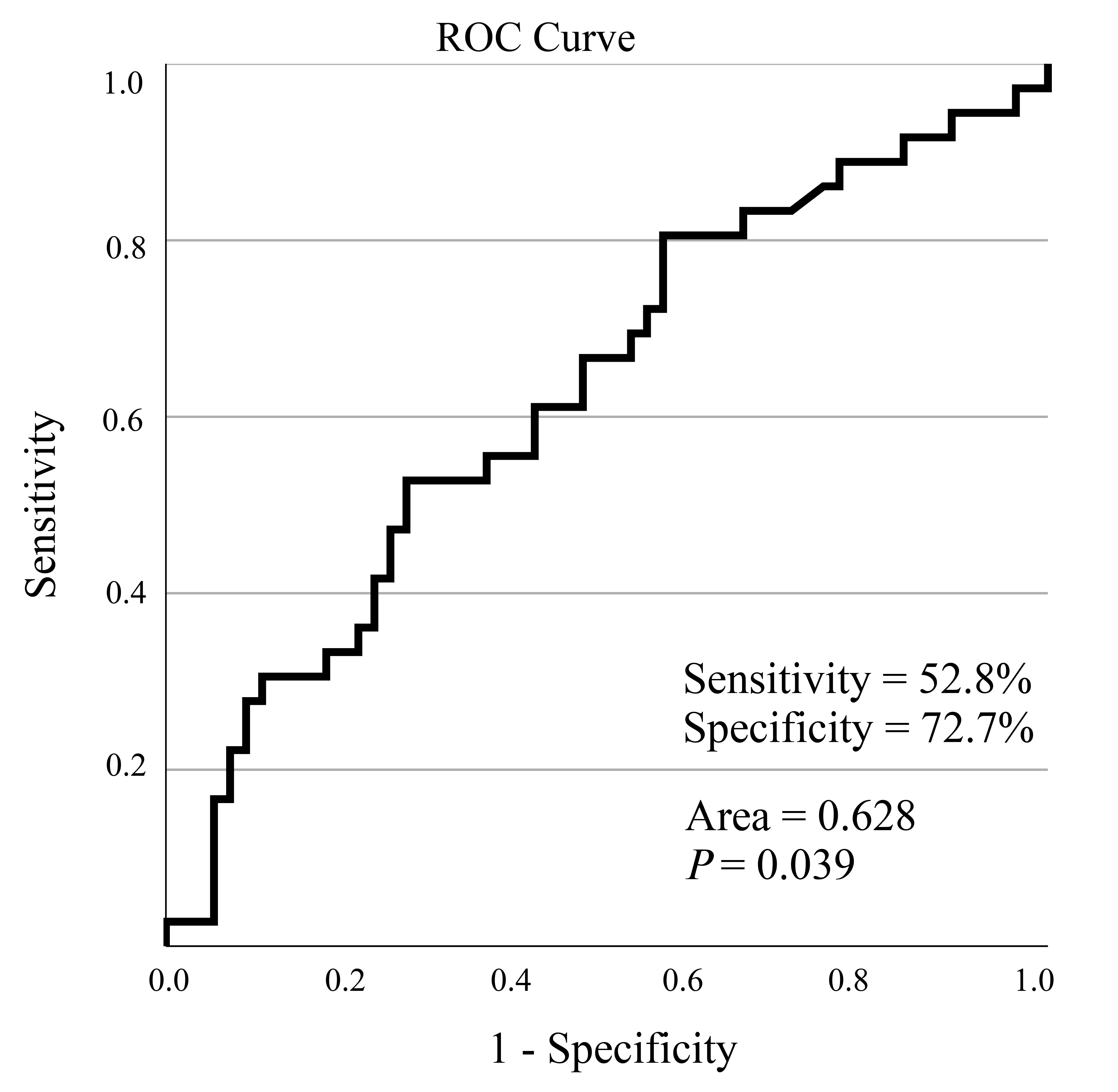

Supplement: Supplementary file 2 — Supplementary file2: ROC Curve of serum AFP values (TIF 498 KB) [file 10120_2025_1594_MOESM2_ESM.tif]

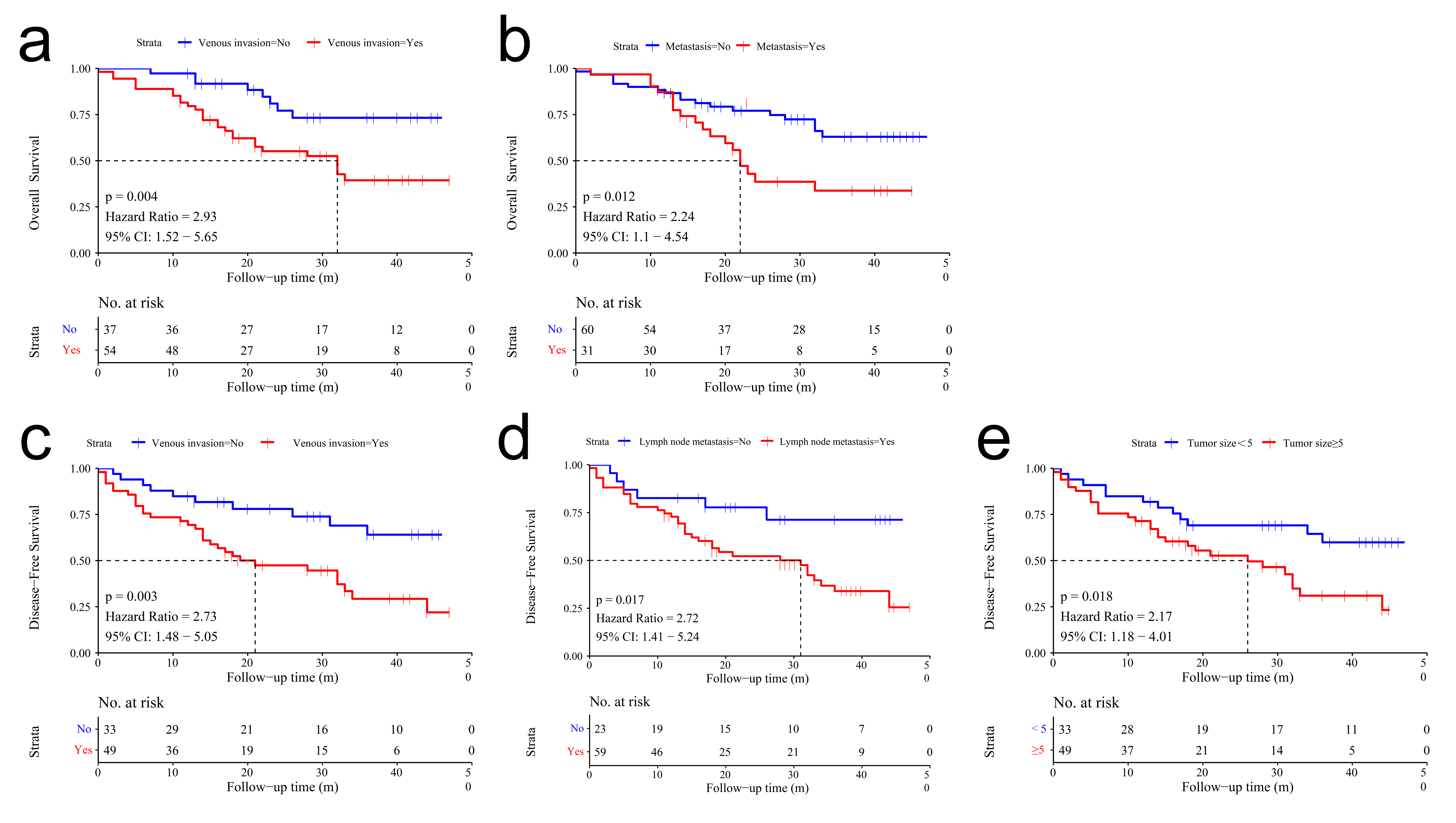

Supplement: Supplementary file 3 — Supplementary file3: Kaplan–Meier curves for overall survival (OS) and Disease-Free Survival (DFS). a, b: Patients with venous invasion, and metastasis had poor OS. c–e: Patients with venous invasion, lymph node metastasis, and tumor size ≥ 5 cm had poor DFS (TIF 13908 KB) [file 10120_2025_1594_MOESM3_ESM.tif]
