# Supplementary material for: Genomic landscape and potential therapeutic targets in alpha-fetoprotein-producing gastric cancer
Source: Gastric Cancer. 2025 Feb 10;28(3):372–83. doi: 10.1007/s10120-025-01594-x (PMC11993487; doi:10.1007/s10120-025-01594-x)
Supplement: Supplementary file 4 — (DOCX 28 KB) [file 10120_2025_1594_MOESM4_ESM.docx]

**Table 1** **Comprehensive clinicopathological characteristics of different subtypes of 91 AFPGC patients**

| Clinical characteristic | COM | ENT | HPT | YST | Total | *P*-value |
| --- | --- | --- | --- | --- | --- | --- |
| Sex |  |  |  |  |  | 0.740 |
| Male | 28 | 11 | 31 | 3 | 73 |  |
| Female | 7 | 1 | 9 | 1 | 18 |  |
| Age (yr) |  |  |  |  |  | 0.275 |
| <60 | 10 | 4 | 16 | 3 | 33 |  |
| ≥ 60 | 25 | 8 | 24 | 1 | 58 |  |
| Serum AFP (ng/mL) |  |  |  |  |  | 0.308 |
| <500 | 22 | 10 | 22 | 2 | 56 |  |
| ≥ 500 | 13 | 2 | 18 | 2 | 35 |  |
| Tumor site |  |  |  |  |  | 0.562 |
| Cardia | 16 | 7 | 17 | 1 | 41 |  |
| Gastric antrum | 12 | 5 | 13 | 1 | 31 |  |
| Gastric body | 4 | 0 | 8 | 2 | 14 |  |
| Esophagogastric junction | 3 | 0 | 2 | 0 | 5 |  |
| Depth of invasion |  |  |  |  |  | 0.843 |
| T1-T2 | 7 | 1 | 7 | 0 | 15 |  |
| T3-T4 | 28 | 11 | 33 | 4 | 76 |  |
| pTNM stage |  |  |  |  |  | 0.514 |
| Ⅰ-Ⅱ | 19 | 4 | 19 | 1 | 43 |  |
| Ⅲ-Ⅳ | 16 | 8 | 21 | 3 | 48 |  |
| Tumor size (cm) |  |  |  |  |  | 0.333 |
| <5 | 15 | 4 | 19 | 0 | 38 |  |
| ≥ 5 | 20 | 8 | 21 | 4 | 53 |  |
| Lymph node metastasis |  |  |  |  |  | 0.288 |
| No | 13 | 1 | 11 | 1 | 26 |  |
| Yes | 22 | 11 | 29 | 3 | 65 |  |
| Nerve invasion |  |  |  |  |  | 0.412 |
| No | 24 | 5 | 26 | 3 | 58 |  |
| Yes | 11 | 7 | 14 | 1 | 33 |  |
| Venous invasion |  |  |  |  |  | 0.293 |
| No | 18 | 3 | 14 | 2 | 37 |  |
| Yes | 17 | 9 | 26 | 2 | 54 |  |
| Differentiation |  |  |  |  |  | 0.162 |
| Poorly differentiated | 13 | 3 | 21 | 2 | 39 |  |
| Moderately - Poorly differentiated | 11 | 7 | 15 | 2 | 35 |  |
| Moderately differentiated | 11 | 2 | 4 | 0 | 17 |  |
| AFP |  |  |  |  |  | 0.044* |
| （+） | 13 | 5 | 27 | 2 | 47 |  |
| （-） | 22 | 7 | 13 | 2 | 44 |  |
| GPC3 |  |  |  |  |  | 0.025* |
| （+） | 20 | 6 | 32 | 1 | 59 |  |
| （-） | 15 | 6 | 8 | 3 | 32 |  |
| SALL4 |  |  |  |  |  | 0.008** |
| （+） | 16 | 4 | 29 | 4 | 53 |  |
| （-） | 19 | 8 | 11 | 0 | 38 |  |
| CDX-2 |  |  |  |  |  | 0.505 |
| （+） | 33 | 10 | 38 | 4 | 85 |  |
| （-） | 2 | 2 | 2 | 0 | 6 |  |
| CD10 |  |  |  |  |  | 0.328 |
| （+） | 31 | 9 | 37 | 4 | 81 |  |
| （-） | 4 | 3 | 3 | 0 | 10 |  |
| CEA |  |  |  |  |  | 0.577 |
| （+） | 33 | 10 | 37 | 4 | 84 |  |
| （-） | 2 | 2 | 3 | 0 | 7 |  |
| ATBF1 |  |  |  |  |  | 0.010* |
| （+） | 9 | 4 | 8 | 1 | 22 |  |
| （-） | 26 | 8 | 32 | 3 | 69 |  |
| HNF-1β |  |  |  |  |  | 0.843 |
| （+） | 31 | 10 | 33 | 4 | 78 |  |
| （-） | 4 | 2 | 7 | 0 | 13 |  |
| CLDN6 |  |  |  |  |  | 0.579 |
| （+） | 34 | 11 | 39 | 4 | 88 |  |
| （-） | 1 | 1 | 1 | 0 | 3 |  |
| OCT3/4 |  |  |  |  |  | 0.004 ** |
| （+） | 0 | 3 | 0 | 0 | 3 |  |
| （-） | 35 | 9 | 40 | 4 | 88 |  |
| p53 |  |  |  |  |  | 0.692 |
| Mutant type | 30 | 9 | 32 | 4 | 75 |  |
| Wild type | 5 | 3 | 8 | 0 | 16 |  |
| Her-2 |  |  |  |  |  | 0.577 |
| No amplification | 33 | 10 | 37 | 4 | 84 |  |
| Amplification | 2 | 2 | 3 | 0 | 7 |  |
| Microsatellite |  |  |  |  |  | 0.667 |
| dMMR | 4 | 1 | 8 | 0 | 13 |  |
| pMMR | 31 | 11 | 32 | 4 | 78 |  |

Note: *，*P*<0.05; **，*P*<0.01; ***，*P*<0.001
