# Supplementary material for: Genomic landscape and potential therapeutic targets in alpha-fetoprotein-producing gastric cancer
Source: Gastric Cancer. 2025 Feb 10;28(3):372–83. doi: 10.1007/s10120-025-01594-x (PMC11993487; doi:10.1007/s10120-025-01594-x)
Supplement: Supplementary file 6 — (DOCX 21 KB) [file 10120_2025_1594_MOESM6_ESM.docx]

**Table 3 Multivariate Cox analysis of factors influencing disease-free survival time**

| Factor | HR | 95%CI | | *P*-value |
| --- | --- | --- | --- | --- |
| Tumor size (cm) |  |  |  |  |
| <5cm | - | - | - | 0.304 |
| ≥ 5cm |  |  |  |  |
| Clinical stage |  |  |  |  |
| Ⅰ | 3.041 | 1.630 | 5.674 | <0.001 |
| Ⅱ |  |  |  |  |
| Ⅲ |  |  |  |  |
| Ⅳ |  |  |  |  |
| Tumor site |  |  |  |  |
| Cardia | - | - | **-** | 0.600 |
| Gastric antrum |  |  |  |  |
| Gastric body |  |  |  |  |
| Esophagogastric junction |  |  |  |  |
| Venous invasion |  |  |  |  |
| No | - | - | **-** | 0.178 |
| Yes |  |  |  |  |
| Nerve invasion |  |  |  |  |
| No | 2.917 | 1.532 | 5.594 | 0.001 |
| Yes |  |  |  |  |
| Lymph node metastasis |  |  |  |  |
| No | - | - | **-** | 0.775 |
| Yes |  |  |  |  |
| Serum AFP (ng/mL) |  |  |  |  |
| <500 | 1.998 | 1.035 | 3.857 | 0.039 |
| ≥ 500 |  |  |  |  |
| ARID1A |  |  |  |  |
| Wild-type | - | - | **-** | 0.979 |
| Mutant |  |  |  |  |

CI, confidence interval; HR, hazard ratio.
